# Demonstrative Midwifery

**Published:** 1851-02

**Authors:** 


### *Demonstrative Midwifery.*

In the Dec. No. of this Jonrnal, we charged a review of the Report of the trial of Loomis for libel, signed C. M., in the American Journal of Medical Sciences, No. for Oct., 1850, with containing, first, a misrepresentation of the published facts relating to demonstrative instruction in midwifery at the Medical College in this city; and, second, an unjust imputation of unskillful management of the case of labor which was the subject of demonstration. We pledged ourselves to make good these charges, if occasion seemed to require it.

In the January No. of the American Journal of Medical Sciences, the Editor, premising that he has received a letter from Prof. White respecting the two grounds of complaint just mentioned, and expressing a desire that on every occasion the fullest justice should be done to all persons, says, with regard to the first point, i. e., the misrepresentation of facts, "that the reviewer has honestly expressed the impressions made upon his own mind from a careful perusal of the evidence, and that as he has been misled, others may be equally so; and," he adds, "we are therefore happy to have the opportunity of allowing Professor White to place the facts in their true light."

That any reader, "after a careful perusal of the evidence," should have received the impressions expressed by C. M., is a matter of wonder, but we have no disposition to gainsay the assertion. An individual cannot be held responsible for a lack of apprehension, but it is certainly not an unreasonable ground of complaint that a writer should assume to exercise the functions of a critic, if he be so liable to be misled in the endeavor to comprehend plain facts set forth in a publication which he undertakes to review. In the libel suit growing out of the demonstration of labor at the Buffalo Medical College, inasmuch as the chief object of the prosecution was to have the facts fully and clearly developed, special pains were taken that there should be no occasion for honest misapprehension on this score.

With respect to the management of the case of labor, the position of the head of the child, as ascertained by stethoscopic examinations prior to the delivery, and confirmed by the ocular demonstration, was "occiput to the right posteriorly, face left anteriorly." The Reviewer, C. M., declared a "*great error*" to have been committed in not rectifying this faulty position in the early stage of labor, "by bringing the occiput into such a position as would permit it to emerge under the arch of the pubis, while the face followed the curvature of the sacrum." The Reviewer farther affirmed, that, by omitting this "first duty of an accoucheur," the professor "permitted the life of both mother and child to be put in jeopardy." Concerning this accusation the Editor of the Am. Journal, in the Jan. No., says, "upon consideration of the subject we cheerfully allow that the language of the reviewer was too strong. We consider the practice advocated by Dr. Denman, and the late Prof. Dewees, than whom we cannot admit any higher authority in midwifery, to correct this presentation in the early stage by bringing the occiput into such a position as will permit it to emerge under the arch of the pubis, to be the best; but it must be admitted that the observations of Naegele show that nature herself will often accomplish this, and even when she fails to do so we have several modern authorities for non-interference, and who maintain that though the labor is more protracted, still delivery may take place wlth safety to mother or child."

Were we to discuss the subject in its scientific bearings, we should respectfully dissent from the view, as thus expressed, of the weight of authority respecting the duty of the accoucheur in cases of the position referred to.

We are aware that Denman, Dewees, and other of the older writers on midwifery, recommended early manual interference in such cases, but we suppose, agreeably to views now generally entertained, these eminent writers to have been deceived with respect to the feasibility of the manœuvre recommended by them, attributing, incorrectly, to the efforts of traction exerted upon the head, spontaneous changes effected by the action of the uterus upon the body of the child; and that not only *several*, but the *majority* of the most approved teachers of the present day do not recommend that procedure, by omitting which, according to C. M., a "*great error*" was committed by Prof. White, jeopardising, thereby, the life of mother and child. Be this as it may, the falsity of the accusation of malpractice made by the reviewer with so much assurance, is repudiated by the able Editor of the American journal, in a manner creditable to his honor, and sense of Justice. We cannot, however, avoid expressing surprise that the amende was relinquished by the author of the review to the honor and sense of justice of the Editor. One might not unnaturally be led to presume that a degree of sensibility to be shocked by an ocular demonstration of labor for purposes of instruction, would be accompanied by a corresponding acuteness of the moral sense which would insist upon the privilege of retracting a false accusation into which the writer might, through prejudice or inadvertence, have been betrayed—more especially when the accusation affected a fellow member of the profession, and a public teacher, in a point in which he might well be expected to be most sensitive, viz: his professional knowledge and ability.

We cannot concur with the respected Editor of the American Journal of Med. Sciences in the remark that, inasmuch as these are matters of importance only that injustice be not done to individuals, they are therefore of secondary interest. The rights, the character, and the feelings of individuals, as it seems to us, should be not less sacred than the claims of science and education. We conceive that a writer is in no instance justified in proclaiming with his pen, what would be in violation of propriety and courtesy if pronounced with his tongue. The reviewer and the Editor, we submit, are bound to be guided by the same ethical rules, in the columns of a Journal, which should govern colloquial intercourse. Nay, the moral obligation is even stronger, inasmuch as more injury may be inflicted by written, than by spoken injustice. Who would not choose to abide a verbal slander, circulating slowly, and within circumscribed limits, from mouth to mouth, rather than an aspersion made indelible by print, and scattered n all directions, where the vindication might perhaps never follow it. It will not surely be questioned that for a medical practitioner to stigmatize the practice of his professional neighbor as unskillful, and dangerous to lives, would, among honorable men, be deemed, to say the least, ungenerous, even if there were valid grounds for the charge; but if the imputation were wholly without foundation, it would imply something more than a want of generosity, and could hardly escape harsher terms of rebuke. Now, is it any the less censurable that an unfounded accusation of malpractice is made in the columns of a Journal supposed to circulate more extensively than any other in the United States? If we are mistaken in the belief that there does exist no real distinction in the two cases—that the position of a reviewer furnishes no exemption from those rules of honor, of courtesy, and morality, which should regulate verbal expressions affecting the character or feelings of members of the same profession, then we not only stand ready to be corrected, but to make all proper apologies for any injustice which may be implied in the views we have just expressed.

It would be unfair to close this article without stating that the Editor of the Am. Jour. of Med. Sciences declares his full concurrence with the reviewer in the opinions of the latter with regard to the propriety and advantage of demonstrative midwifery. He expresses also the opinion that these sentiments are not confined to a small minority of the profession. He says, "We have yet to meet with a single respectable physician in Philadelphia who does not concur in them," and they are the sentiments, he adds, "if we may judge from our sources of information, of a large majority of the profession in the United States." The opportunity of the Editor to become conversant with the opinions of the medical profession in Philadelphia, on a point of science or education, it would be presumed, of course, should be better than our own, yet we chance to know that more than one member of the profession of that city, occupying positions second to none, are far from being opposed to demonstrative midwifery. What the editor's sources of information are, with respect to the prevailing sentiment on the subject throughout the United States, we do not assume to know; but the impression derived from our sources of information is quite the reverse of that entertained by him. The discussion of this point, however, would be alike unprofitable and irrelevant. We believe, after considerable reflection, that the interests of science, education, and humanity would be promoted by rendering the study of midwifery demonstrative with the aid of the living subject. This opinion is formed irrespective of the opinions of others, upon a consideration of the merits of the question, uninfluenced, so far as we may judge, by any feeling, or bias. We claim, of course, the prerogative to hold our own opinions on this, as on other topics, and to utter them as we deem proper, at the same time conceding freely to others the same privileges, and desirous, at all times, to treat the views of those who may honestly differ from us, with due consideration and respect. For ourselves, and, we will venture to add, on behalf of the school with which we are connected, it is far from being contended that the profession of the country shall unanimously consider demonstrative teaching in midwifery either expedient, or useful. All that has ever been claimed is, that the facts relating to the subject should not be misrepresented, and ha t motives should be construed with a proper regard to truth and justice.

